# Associations of Duration of Preadoption Out-of-home Care, Genetic Risk for Schizophrenia Spectrum Disorders and Adoptive Family Functioning with Later Psychiatric Disorders of Adoptees

**DOI:** 10.1007/s10578-022-01411-x

**Published:** 2022-08-13

**Authors:** Toni Myllyaho, Virva Siira, Karl-Erik Wahlberg, Helinä Hakko, Tiina Taka-Eilola, Kristian Läksy, Ville Tikkanen, Riikka Roisko, Mika Niemelä, Sami Räsänen

**Affiliations:** 1 Unit of Clinical Neuroscience, Psychiatry, University of Oulu, P.O. Box 5000, 90014 Oulu, Finland; 2 Faculty of Education, University of Oulu, P.O. Box 2000, 90014 Oulu, Finland; 3 Department of Psychiatry, Oulu University Hospital, P.O. Box 26, 90014 Oulu, Finland; 4 Faculty of Medicine, Center for Life Course Health Research, University of Oulu, P.O. Box 5000, 90014 Oulu, Finland; 5 Faculty of Medicine, Research Unit of Clinical Neuroscience, Psychiatry, University of Oulu, P.O. Box 5000, 90014 Oulu, Finland; 6 Department of Psychiatry, Basic Health Care District of Kallio, Oulu, Finland

**Keywords:** out-of-home care, genetic risk, schizophrenia spectrum disorders, gene-environment interaction, adoptive family functioning

## Abstract

The objective was to examine the impacts of duration of preadoption out-of-home care and adoptive family functioning on later psychiatric morbidity of adoptees with high (HR) and low (LR) genetic risk for schizophrenia spectrum disorders. The study uses nationwide data from the Finnish Adoptive Family Study of Schizophrenia. The study population in this substudy consisted of 43 h adoptees and 128 LR adoptees. Of these adoptees, 90 had spent 0–6 months and 81 over 6 months in preadoption out-of-home care. The family functioning of adoptive families was assessed based on Global Family Ratings and psychiatric disorders on DSM-III-R criteria. The results showed that among the adoptees with over 6 months in preadoption out-of-home care, the likelihood for psychiatric disorders was significantly increased in HR adoptees compared to LR adoptees. In adoptees with 6 months or less in preadoption out-of-home care, an increased likelihood for psychiatric disorders was found among those living in adoptive families with dysfunctional processes. These findings indicate that especially for HR children, a well-functioning early caregiving environment is crucial in terms of subsequent mental wellbeing. The results emphasize that when adoption is necessary, early placement and well-functioning adoptive family environment are beneficial to children.

## Introduction

It has been estimated that approximately 3.2 million children are placed in out-of-home care worldwide yearly [1]. Studies have reported that in the U.S., about 660,000 children [2] and in the EU, nearly one million children [3] experience out-of-home care placement every year. In Finland, approximately 1.1% of all the children in the population are placed or live in out-of-home care every year, and of infants aged 0–2 years, 0.2% were in out-of-home care in the year 2019 [4]. Heino et al. [5] reported that in Finland, the main reasons for placement in out-of-home care are parents’ mental health problems (33%) and substance abuse (26%). Likewise, parental mental health problems and substance use, especially those of biological mothers, have been reported to increase the risk for out-of-home care for offspring [6].

Previous research has documented that children of mothers with schizophrenia are at 12.6–23.75 times higher (incidence rate ratio = IRR) risk of being placed in out-of-home care during their infancy and childhood compared to children of mothers from general population [7, 8]. The study of Ranning et al. [7] showed that the risk of being placed in out-of-home care is particularly high (IRR = 80.2) during the child’s first year for children of mothers with schizophrenia. It has been reported that around half of the mothers with schizophrenia lose custody of their children either temporarily or permanently [9], which predisposes children to out-of-home care and also adoption.

Preadoption out-of-home care, such as institutional or foster care, which lasts over 6 or 12 months [10–14] has been found to cause instability and disruptions in early caregiving [14] and increase the risk for psychiatric care in adulthood [15]. Institutional rearing has been considered to be a suboptimal caregiving environment for young children [16–19] due to features such as low levels of caregiver-child interaction and shifting caregivers [18, 19]. The nature of institutional rearing is also proposed to weaken children’s opportunities to form attachment relationships with caregivers [20].

According to attachment theory [21, 22], by the end of the first year of life, most infants develop an attachment towards the early caregiver, which is shown to be dependent upon the quality of the received care [12, 23]. Early attachment relationships have been documented to be important not only for the development of stress and affect regulation capacities [24] but also for the infant’s neurobiological development [25–28].

Genome-wide association studies (GWAS) have shown that many of the genetic variants that associate with the development of schizophrenia are related to early pre- and perinatal neurodevelopment [29, 30]. Studies have reported that early perinatal adversities contribute to the development of schizophrenia [29]. The neurodevelopmental models of schizophrenia have proposed that early developmental insults interact with genetic factors in aberrant brain development that may mediate the risk for the development of schizophrenia [31].

After placement in adoptive families with improved caregiving, adoptees show notable developmental catch-up and recovery following the possible adverse effects of early institutionalization [12, 32–34]. However, although the negative effects of early adverse experiences are shown to attenuate over time [35], not all adopted children show equal developmental catch-up [10] regardless of the time spent in adoptive families [11, 17]. Van IJzendoorn et al. [32] have suggested that the degree of recovery following institutionalization may depend on the characteristics of the adoptive family, such as parental sensitivity or socioeconomic status of the adoptive family. On the other hand, the study of Finet et al. [36] did not find adoptive parenting to moderate the associations between preadoption experiences and children’s behavioral adjustment.

Generally, the research evidence on the role of family functioning in mitigating the possible maladjustments caused by preadoption experiences has so far remained sparse and inconclusive. Although adopted children have been shown to benefit from improved caregiving in adoptive families [32, 33], earlier studies have rarely been able to assess the quality of adoptive families’ functioning. Furthermore, in studies that have focused on the impacts of institutionalization, the genetic background of the adopted children and its impact on the later development have remained unknown, although their importance has been widely acknowledged [12, 27, 37]. The earlier findings from the Finnish Adoptive Family Study of Schizophrenia have shown that the quality of adoptive family functioning, assessed with the Global Family Ratings (GFRs), associates with adoptees’ later psychiatric morbidity, especially in adoptees with high genetic risk for schizophrenia spectrum disorders [38–40]. However, in these studies the time in preadoption out-of-home care has not been considered.

In this study, the impacts of genetic risk for schizophrenia spectrum disorders and adoptive family functioning on later psychiatric morbidity of the adoptees were assessed, separately, for those exposed to short (≤ 6 months) and longer (> 6 months) preadoption out-home care. The preadoption out-of-home care was provided by municipal social services. In Finland, institutional care has been typically the most common alternative for out-of-home care and there has been notable differences in the quality of care [41] but unfortunately detailed information was not available. The study used national data from the Finnish Adoptive Family Study of Schizophrenia, that allowed the analysis of genetic liability for schizophrenia spectrum disorders and environmental factors separately in the development of psychiatric disorders.

## Methods

### Subjects

The current study utilizes the nationwide data from the Finnish Adoptive Family Study of Schizophrenia. The study design has been described in detail elsewhere [42–44] and it is addressed here shortly. In the study design, the adoptees who are not reared by their biological parents, are examined to identify the genetic (schizophrenia liability) and environmental contributions (rearing environment), as well as their interaction, in the development of psychiatric disorders. Initially, the population of the study was based on the hospital records that covered all the women (n = 19,447) who were admitted to psychiatric care in Finnish hospitals during the years 1960 ÷ 1979. After that, the women who had been diagnosed at least once with schizophrenia or paranoid psychosis were identified from this study population. These women were further scrutinized through census and parish registers with the aim of identifying the ones who had given up a child or children (high-risk adoptees) for adoption. The adoptees who were adopted after the age of four, adopted abroad or were adopted by relatives were excluded from the study population. No diagnostic exclusion criteria were applied to adoptive parents and therefore they represent an epidemiological sample of adoptive parents 42, 44].

The high-risk adoptees (HR) and their adoptive families were demographically matched with low-risk (LR) control adoptees and their adoptive parents. The control adoptees, to be scrutinized further, were selected among those who were given up for adoption by biological mothers who in the primary phase of the study either had no psychiatric diagnosis or were diagnosed with a psychiatric disorder other than a schizophrenia spectrum disorder. The matching criteria included sex and age of the adoptee, the age of the adoptee at the time of the placement, and socioeconomic status of the adoptive family [42, 43]. The adoptive families of the study were assessed by experienced psychiatrists with broad research procedures which included family observations, interviews and psychological tests both individually and with different family combinations [42].

The diagnoses of all biological mothers were verified. Broad schizophrenia spectrum disorders (DSM-III-R) [45] comprised the following disorders according to Kendler et al. [46]: schizophrenia, the odd-cluster personality disorders (schizotypal, schizoid and paranoid personality disorders plus avoidant personality disorder), non-schizophrenic non-affective psychoses (schizoaffective, schizophreniform, and delusional disorders and psychotic disorder not otherwise specified) and affective psychoses (bipolar and depressive disorders with psychotic features) [43].

The adoptees were defined to have HR for schizophrenia spectrum disorders, if they were given up for adoption by a mother with a verified diagnosis of a broad schizophrenia spectrum disorder. The LR adoptees had a biological mother who had either a non-spectrum diagnosis or no psychiatric diagnosis [42]. The final study population (n = 382) of the Finnish Adoptive Family Study of Schizophrenia was comprised of 190 adoptees at HR and 192 adoptees at LR for schizophrenia spectrum disorders [42, 44].

The current study sample consists of 171 adoptees (43 h adoptees, 128 LR adoptees) and their adoptive families for whom the information of time in preadoption out-of-home care, family functioning and time with biological mother was available for statistical analyses.

An attrition analysis (Table S1, available online) was conducted to examine whether the current study sample (n = 171) differed from those not included in the analyses. The current study sample differed statistically significantly from the not-included adoptees with regard to their genetic status (HR adoptees: 25.1% vs. 69.7%, p < 0.001) and time spent with biological mother (one month or more, 49.7% vs. 30.6%, p = 0.010).

### Measures

#### Psychiatric disorders of the adoptees

The adoptees’ psychiatric disorders were based on DSM-III-R [45] diagnostic classification and its criteria, which were in use when the research diagnoses were verified. DSM-III-R is a descriptive classification system, compatible with the current DSM-5 version [47], and their criteria are mainly similar. The category of schizophrenia spectrum disorders included diagnoses for schizophrenia, the odd-cluster personality disorders (schizotypal, schizoid and paranoid personality disorders plus avoidant personality disorder), non-schizophrenic non-affective psychoses (schizoaffective, schizophreniform, and delusional disorders and psychotic disorder not otherwise specified), and affective psychoses (bipolar and depressive disorders with psychotic features). The psychiatric disorders other than schizophrenia spectrum disorders included all the non-schizophrenia spectrum disorders [43].

The DSM-III-R diagnoses of the adoptees were made by personal interviews and from hospital records and other diagnostically significant sources available [43]. At the time of the initial assessment, the adoptees’ median age was 23 years (IQR 17–33 years). The final diagnostic evaluation of the adoptees took place 21 years after the initial assessment when adoptees’ median age was 44 years (IQR 38–52). The psychiatrists who made the diagnostic evaluations were blinded to the adoptees’ genetic risk status and prior psychiatric evaluations. The psychiatric status of the adoptees was defined as the hierarchically most severe lifetime diagnosis [40, 43, 46]. The kappa coefficient for interrater reliability of the adoptees’ diagnoses was 0.71–0.80 [42].

In this study, the adoptees’ psychiatric disorders were classified into two categories: (1) any diagnosed psychiatric disorder (both schizophrenia spectrum disorders and other than schizophrenia spectrum disorders) and (2) no psychiatric diagnosis. The classification was based on the accumulating evidence that genetic liability for schizophrenia increases the risk not only for schizophrenia but also for other psychiatric disorders in the offspring of a parent with schizophrenia [48, 49]. Also, many adverse childhood stressors, such as aberrant mother-child interaction and dysfunctional family relationships, are documented to be common precursors for a wide range of psychiatric disorders [50, 51].

#### Preadoption out-of-home care

The time spent in preadoption out-of-home care refers to the time span which the adoptees spent neither with their biological mothers nor in adoptive families. The time in preadoption out-of-home care before placement into the adoptive family was categorized into two groups: (1) 0–6 months and (2) over 6 months. This categorization was based on previous findings showing that, compared to noninstitutionalized children, institutionalization which lasts over six months associates with multiple developmental deficits and attachment-related problems [10, 11].

Among the adoptees who were in preadoption out-of-home care 0–6 months, the median age at the time of the placement in adoptive families was 6 months (sd = 8.3 months; IQR 3.9–10 months). For the adoptees who were in preadoption out-of-home care over 6 months, the median age at the time of the placement in adoptive families was 20 months (sd = 12 months; IQR 14–29 months).

#### Family functioning

This study used Global Family Ratings (GFRs) to assess the broad level of functioning of adoptive families. The method has been described in detail elsewhere [38, 40] and will be discussed here briefly. GFRs were assessed multi-methodically on interviews, observations and tests which measure the functioning of families comprehensively from different perspectives. The experienced researchers conducted the evaluations during the home visits to adoptive families and were blinded to the genetic status of the adoptees. The initial assessments were later re-evaluated and a sample of 40 recorded interviews was rated by three research interviewers to define the reliability of the ratings. The interrater-reliability was regarded reasonable (0.72) on the scale from 0 = poor to 1 = high concordance [40].

Each adoptive family was evaluated on the following factors: (1) Anxiety, (2) Basic trust, (3) Boundaries, (4) Conflicts, (5) Empathy, (6) Flexibility of homeostasis, (7) Interaction and its quality, (8) Parental coalition, (9) Power relations, (10) Reality testing, and 11) Transactional defenses. Initially, the GFRs were used as a basis to classify the families into five categories on a scale from 1) healthy families to 5) severely disturbed and chaotic families [40]. This categorization is based conceptually on the hypothetical continuum of the five-level Global Assessment of Relational Functioning (GARF), published originally in DMS-IV [52]. Contemporary family research literary was used in the formulation of the GFRs categories [53].

Due to significant similarities between the GFRs categories 1 and 2, and categories 4 and 5 [38, 40], the five GFRs categories of family functioning were regrouped into three groups: (1) Families with functional processes (GFRs categories 1 & 2), in which, for example, the levels of anxiety and quality of interaction were considered healthy; (2) Families with mildly dysfunctional processes (GFRs category 3), in which, for example, the levels of anxiety and quality of interaction were estimated as moderately dysfunctional; and (3) Families with dysfunctional processes (GFRs categories 4 & 5), in which, for instance, the levels of anxiety and patterns of interaction were deemed to be detrimental for the family members [38, 40].

#### Time with biological mother

The time with biological mother was also explored. It was categorized into two groups: (1) less than one month with biological mother, (2) one month or above. The aim of this categorization was to explore whether immediate out-of-home placement after birth impacted children differently compared to later placement away from biological mother. The first group of adoptees may also indicate a group of children that were in urgent need for an out-of-home placement because they spent less than a month with their biological mothers.

For the adoptees who were in preadoption out-of-home care 0–6 months, the median time with the biological mother was 3.3 months (sd = 8.8 months; IQR 0-9.3 months). Among the adoptees who were in preadoption out-of-home care over 6 months, the median time with the biological mother was 0 months (sd = 4.6 months; IQR 0-4.5 months).

#### Statistical analyses

Statistical significance of group differences in categorical variables was assessed with Pearson’s Chi-Square test or Fisher’s Exact Test. A logistic regression model was used to examine the association of genetic risk, family functioning (GFRs), gender and time with biological mother with the follow-up diagnosis of any psychiatric disorder of the adoptees, separately, for the two preadoption out-of-home care groups (0–6 months, over 6 months). An additional exploratory analysis (Table S2, available online) was conducted to explore the bivariate association between adoptive family functioning and psychiatric morbidity of the adoptees in the data stratified both by the length of preadoption out-of-home care and genetic risk for schizophrenia spectrum disorders.

A sensitivity analysis was performed to assess the robustness of the findings based on the categorization of the adoptees into two groups according to the preadoption out-of-home care time (≤ 6 months, > 6 months). In the sensitivity analysis (Table S3, available online), the cut-off value of 12 months for preadoption out-of-home care time was used to stratify the adoptees into two groups (≤ 12 months, > 12 months). The choice of 12 months as a cut-off time was based on earlier studies which have suggested that institutionalization may require a longer time to have detrimental impacts on children’s development [11, 54].

All tests were two-tailed and the limit for statistical significance was set at p = 0.05. The statistical software used in the analyses was IBM SPSS Statistics Version 26.

## Results

Table 1 presents the characteristics of the adoptees stratified into two groups according to the preadoption out-of-home care time. In both study groups, over 70% of the adoptees belonged to the low risk for schizophrenia spectrum disorders (LR) group. Those adoptees with 6 months or less in preadoption out-of-home care spent more time with their biological mother, whereas among the adoptees over 6 months in preadoption out-of-home care spent shorter time with biological mother (p = 0.011).

**Table 1:** Characteristic of the adoptees in relation to preadoption out-of-home care time

Characteristics	Total sample (n = 171)	In preadoption out-of-home care		
		0–6 months (n = 90)	over 6 months (n = 81)	p-value
Genetic risk for schizophrenia spectrum disorders High Risk Low risk	43 (25.1%) 128 (74.9%)	22 (24.4%) 68 (75.6%)	21 (25.9%) 60 (74.1%)	0.824
Family functioning Functional processes Mildly dysfunctional processes Dysfunctional processes	72 (42.1%) 55 (32.2%) 44 (25.7%)	41 (45.6%) 25 (27.8%) 24 (26.7%)	31 (38.3%) 30 (37%) 20 (24.7%)	0.419
Gender Male Female	75 (43.9%) 96 (56.1%)	36 (40%) 54 (60%)	39 (48.1%) 42 (51.9%)	0.284
Time with biological mother in months 0 month 1 month or more	86 (50.3%) 85 (49.7%)	37 (41.1%) 53 (58.9%)	49 (60.5%) 32 (39.5%)	0.011
Diagnosed psychiatric disorder Yes No	69 (40.4%) 102 (59.6%)	37 (41.1%) 53 (58.9%)	32 (39.5%) 49 (60.5%)	0.831

Tables 2a and 2b show the characteristics of the adoptees in relation to psychiatric disorders, stratified by the preadoption out-of-home care time. Among the adoptees with over 6 months of preadoption out-of-home care (Table 2b), the likelihood for any psychiatric disorder was significantly increased in HR adoptees (adj. OR 3.12, 95% CI 1.06–9.20) compared to LR adoptees. For the adoptees with 6 months or less of preadoption time (Table 2a), an increased likelihood for any psychiatric disorder was found among those living in an adoptive family with dysfunctional processes (adj. OR 5.09, 95% CI 1.60–16.18).

In an additional exploratory analysis (Table S2, available online), the bivariate association between adoptive family functioning and psychiatric morbidity of the adoptees was further explored in the data stratified both by the length of preadoption out-of-home care and genetic risk for schizophrenia spectrum disorders. The only statistically significant associations were found between adoptive family functioning and psychiatric morbidity in both HR (p = 0.037) and LR adoptees (p = 0.028) in the subgroups with 6 months or less in preadoption out-of-home care (Table S2, available online). In the subgroups of HR (n = 22) adoptees and LR (n = 68) adoptees with 6 months or less in preadoption out-of-home care (Table S2, available online), the prevalence of psychiatric morbidity was significantly (p < 0.05) low in the adoptees raised in adoptive families with functional processes (HR 25%; LR 31%). Among the early placed adoptees who were exposed to dysfunctional processes in the adoptive families, the prevalence of psychiatric morbidity was particularly high (HR 80%; LR 44%). Corresponding results for adoptees over 6 months in preadoption out-of-home care were non-significant.

The results of the sensitivity analysis are presented in the Table S3 (available online). When preadoption out-of-home care time was re-categorized using 12 months as cut-off time (Table S3, available online), in adoptees with 12 months or less of preadoption time (Table S3a, available online), the likelihood for any psychiatric disorder was significantly associated with families with dysfunctional processes (adj. OR 4.15, 95% CI 1.48–11.66) and marginally significantly associated with female gender (adj. OR 2.18, 95% CI 0.95–4.97). In the study group of adoptees with over 12 months in preadoption out-of-home care (Table S3b, available online), the likelihood for any psychiatric disorder was increased among HR adoptees (adj. OR 3.93, 95% CI 1.02–15.08).

**Table 2:** Association of the characteristics of the adoptees with likelihood for psychiatric disorders, by the length of pre-adoption out-of-home time (≤ 6 months, > 6 months)

**a) Pre-adoption out-of-home care time 0–6 months**							
	Total n of cases (n = 90)	Adoptees with any psychiatric disorder				Likelihood for psychiatric disorder	
		Yes (n = 37)	No (n = 53)	p-value		adj. OR*	95% CI
Genetic risk for schizophrenia spectrum disorders Low risk High risk	68 22	26 (38.2%) 11 (50%)	42 (61.8%) 11 (50%)	0.330		ref. 1.85	0.64–5.30
Family functioning Functional processes Mildly dysfunctional processes Dysfunctional processes	41 25 24	12 (29.3%) 9 (36%) 16 (66.7%)	29 (70.7%) 16 (64%) 8 (33.3%)	0.010		ref. 1.36 5.09**	0.46–4.05 1.60-16.18
Gender, Male Female	36 54	13 (36.1%) 24 (44.4%)	23 (63.9%) 30 (55.6%)	0.431		ref. 1.56	0.61–4.01
Time with biological mother in months 0 month 1 months or more	37 53	12 (32.4%) 25 (47.2%)	25 (67.6%) 28 (52.8%)	0.162		ref. 1.22	0.47–3.17
**b) Pre-adoption out-of-home care time over 6 months**							
	Total n of cases (n = 81)	Adoptees with any psychiatric disorder				Likelihood for psychiatric disorder	
		Yes (n = 32)	No (n = 49)	p-value		adj. OR*	95% CI
Genetic risk for schizophrenia spectrum disorders Low risk High risk	60 21	19 (31.7%) 13 (61.9%)	41 (68.3%) 8 (38.1%)	0.015		ref. 3.12**	1.06–9.20
Family functioning Functional processes Mildly dysfunctional processes Dysfunctional processes	31 30 20	11 (35.5%) 13 (43.3%) 8 (40%)	20 (64.5%) 17 (56.7%) 12 (60%)	0.820		ref. 1.52 1.18	0.52-4-52 0.34–4.07
Gender, Male Female	39 42	13 (33.3%) 19 (45.2%)	26 (66.7%) 23 (54.8%)	0.273		ref. 1.43	0.55–3.75
Time with biological mother in months 0 month 1 months or more	49 32	16 (32.7%) 16 (50%)	33 (67.3%) 16 (50%)	0.118		ref. 1.51	0.56–4.03

* Odds ratios (ORs) and 95% CI of OR are based on the logistic regression analysis assessing the likelihood for psychiatric disorder of the adoptees after adjusting for genetic risk, family functioning, gender and time spent with biological mother

** p < 0.05

*** p < 0.1

## Discussion

Early out-of-home care, such as institutional care, is shown to have adverse effects on children’s development and psychological wellbeing [14, 16–19, 54]. However, in this context the impact of genetic factors on the development of psychiatric disorders has remained unexplained, as only a limited number of studies have been able to control the children’s genetic background and rearing environment. In this study, we were able to examine the impacts of the duration of preadoption out-of-home care, apart from the biological mother, on the associations of high (HR) and low (LR) genetic risk for schizophrenia spectrum disorders and adoptive family functioning with the adoptees’ any later psychiatric disorder. This information will facilitate the development of more secure out-of-home care for children of mothers with a schizophrenia spectrum disorder who are not able to foster their children.

This study has two main findings. The first one is that HR for schizophrenia spectrum disorders was found to associate with increased risk for any later psychiatric disorder in the adoptees with over 6 months in preadoption out-of-home care. This may indicate that, compared to adoptees with LR for schizophrenia spectrum disorders, HR adoptees are especially vulnerable to deficiencies and instability in early caregiving. Indeed, many of the genetic variants that associate with the development of schizophrenia are related to early neurodevelopment [29, 30]. Furthermore, in the neurodevelopmental models of schizophrenia, early developmental insults have been suggested to interact with genetic factors to produce deviant brain development which enhance the risk for the development of schizophrenia [31].

This finding supports the earlier studies that have discussed the role of genetic risk and gene-environment interaction as explaining factors for the outcomes of institutional rearing and inadequate early caregiving [12, 27, 37]. Furthermore, it is also possible that the LR adoptees with prolonged stays in out-of-home care were more resilient than HR adoptees towards early instable and possibly deficient caregiving. Unfortunately, our data lacked more detailed information to confirm this plausible explanation. Earlier studies have suggested that some adoptees show extensive resilience in early adversities [55].

Furthermore, among these adoptees with over 6 months in preadoption out-of-home care, the subsequent psychiatric morbidity did not associate with adoptive family functioning. In our study the adoptees who were in preadoption out-of-home care 0–6 months were placed in adoptive families at the median age of 6 months, whereas the adoptees who were in preadoption out-of-home care over 6 months came in adoptive families at the median age of 20 months. Tottenham [19] has suggested that instable early caregiving, such as institutional care, may preclude children from forming an early attachment to any specific caregiver during the sensitive phase between 6 and 12 months of age. Also, later age at adoption is argued to complicate the attachment processes between the adopted child and adoptive parents [14, 56], and impair children’s ability to respond to new changing caregiving environments when adopted [18–20]. Although positive characteristics of the adoptive family, such as sensitive parenting, may enhance adoptees’ later development [32], it has been noted that improved caregiving in adoptive families may not be sufficient to reduce some of the deviant behaviors that the adoptees may have adapted to during the long preadoption period [16].

Consequently, the second main finding of this study is that when the duration of preadoption out-of-home care was 6 months or less, adoptees’ subsequent psychiatric morbidity was associated with the functioning of the adoptive families. Our results showed that among the adoptees with 6 months or less in preadoption out-of-home care, the dysfunctional family processes in adoptive families, but not genetic risk, *per se*, were associated with an increased likelihood of any later psychiatric disorder of the adoptees. The results of the additional exploratory bivariate analysis (Table S2, available online) showed significant associations between adoptive family functioning and psychiatric morbidity in both HR and LR adoptees in the subgroups with 6 months or less in preadoption out-of-home care. These findings emphasize further the role of the early caregiving environment in modifying the trajectory of children’s development, which is prominent especially for the adoptees with high genetic risk for schizophrenia spectrum disorders. Also, this finding may indicate that early well-functioning caregiving environment can be protective against later psychiatric morbidity for both HR and LR adoptees.

Thus, it may be possible that for adoptees who spent only 6 months or less in out-of-home care before permanent placement, the functioning of the adoptive families at least partially attenuated the negative effects of the adoptees’ genetic background. Knudsen et al. [26, 21, 22] have suggested that humans are most sensitive to environmental influences during the early infancy. This could explain why the high risk for schizophrenia spectrum disorders, *per se*, did not associate with psychiatric morbidity among the adoptees with 6 months or less in preadoption out-of-home care, since their development was influenced significantly by the functioning of the adoptive family. The neurobiological development of humans is shown to be both genetically-driven and experience (environment)-dependent [26, 28], the quality and stability of early caregiving being of great importance [25].

This study used national data from the Finnish Adoptive Family Study of Schizophrenia, which enabled the examination of genetic and rearing environment factors separately [42–44]. This is to be considered a major strength for this study, as the data offers a unique opportunity to examine the impacts of genetic and environmental causes in the development of psychiatric disorders. Although it is plausible that the adopted children also had an impact on the functioning of the adoptive families, the earlier studies from the Finnish Adoptive Family Study of Schizophrenia have demonstrated that the HR adoptees are not the cause of dysfunctional processes in the adoptive families [57]. Also, there was no diagnostic exclusion criteria applied to the adoptive parents. This is to be considered as a strength for our study as the adoptive parents represent an epidemiological, diagnostically normal demographic sample.

Furthermore, with the fine-grained adjustment of family functioning (GFRs) [38, 40] we were able to clarify the role of family functioning and its associations with adoptees’ psychiatric status when the duration of preadoption out-home-care was considered. The adoptive families and adopted children were met and interviewed to examine the family functioning and diagnostic status, which earlier studies have not done this thoroughly. The GFRs are comprehensive evaluations of adoptive family functioning and may therefore represent a clustered risk score, which some studies have preferred to be utilized when examining the impacts of environmental adversities [58]. It can be possible that in the families in which there were more dysfunctional family processes, there were also more substance abuse and other adversities. Also, it is probable that adoptive parents’ possible psychiatric disorders contributed to the ratings of family functioning.

A significant limitation of this study is that we cannot elucidate the quality of the preadoption out-of-home care that was organized by social services and this has to be considered in the interpretation of the results. In Finland, institutional care has been the most common option for out-of-home care although there have been notable municipal differences in child protection services [41]. Furthermore, it has been stated that in so-called globally depriving institutions, detrimental effects on children’s development may occur in less time compared to more adequate institutions [17]. Thus, it is possible that the chosen cut-off point for the outcome variable (≤ 6 months and > 6 months in out-of-home care) is not optimal. To assess the used cut-off point and our findings, we performed a sensitivity analysis (Table S3, available online) with a different cut-off value (≤ 12 months, > 12 months). The sensitivity analysis showed that extended duration of preadoption out-of-home care (because of later cut-off point) had an effect on the associations of genetic risk for schizophrenia spectrum disorders in the groups with longer preadoption time (6 months vs. > 12 months, OR 3.12 vs. OR 3.93) and adoptive family functioning in the groups with shorter preadoption time (≤ 6 months vs. ≤ 12 months, OR 5.09 vs. OR 4.15) with adoptees’ later psychiatric morbidity. The analysis therefore indicates that the impact of adoptees’ genetic background on the risk for any later psychiatric disorder is more pronounced as the time in preadoption out-of-home care progresses. Moreover, extended time in preadoption out-of-home care seems to weaken the impact of the adoptive family’s functioning on adoptees’ later psychiatric morbidity. Thus, the sensitivity analysis supports our initial findings.

Furthermore, the attrition analysis (Table S1, available online) showed that the current sample differed significantly from the non-included adoptees with regard to genetic risk status for schizophrenia spectrum disorders (p = < 0.001) and time with biological mother (p = 0.010). In the current study, HR represented only 25.1% of the adoptees, compared to 50% in the total data. It may be possible that the dysfunctional adoptive families with HR adoptees were less willing to participate in the study, since not only are there less HR adoptees than LR adoptees in the study sample but there are also less dysfunctional than functional adoptive families in the study sample. Therefore, these circumstances could have affected our results, and particularly in HR adoptees, conclusions must be made with caution. However, it is also possible that our results could be more pronounced if more HR adoptees and their adoptive families had participated in the study.

Although the size of the current study sample in analyses was moderate, lack of statistical power in subgroup analyses (type 2 error) may have occurred. Due to lack of data, we are not able to confirm if the adoptees had multiple placement breakdowns before they were placed permanently in the adoptive families. It has been shown that early placement breakdowns can be detrimental for children’s attachment security [56]. Finally, there is a possibility that the HR children who expressed more abnormal traits and behaviors may have been institutionalized for longer periods [59], which may have impacted our results. It may be possible that the HR children in this study who experienced more extensive out-of-home care expressed some deviant behaviors and because of those, were adopted later.

It is important that future research with larger study populations aim to confirm our findings. Especially, the finding regarding the impacts of genetic liability for schizophrenia spectrum disorders, needs to be confirmed by other studies. Future studies that can elaborate the quality of preadoption out-of-home and also consider adoptees’ genetic background are needed to explain this matter more precisely. However, it is important to emphasize that collecting a nationwide data, similar to ours, would be challenging and also very expensive, which enhances the value of our findings.

Children of mothers with schizophrenia are shown to be at increased risk of being placed in out-of-home care during their infancy and childhood [7, 8]. Therefore, it is critical to develop practices and policies that secure a safe caregiving environment for these genetically vulnerable children. The results can be utilized in developing out-of-home care, foster and adoption practices for children, particularly in high-risk populations. In addition, the results can help to target early interventions during sensitive periods in child development. Furthermore, the results can be utilized in planning family-centered psychosocial support for adoptive families in which the adopted child has experienced preadoption adversities.

### Summary

In summary, this study examined the impacts of duration of preadoption out-of-home care and adoptive family functioning on later psychiatric morbidity of adoptees with high (HR) and low (LR) genetic risk for schizophrenia spectrum disorders. The study used national data from the Finnish Adoptive Family Study of Schizophrenia. The study population in this substudy consisted of 43 h adoptees and 128 LR adoptees of whom 90 were 0–6 months and 81 over 6 months in preadoption out-of-home care. The study used the Global Family Ratings to assess the functioning of adoptive families and DSM-III-R criteria to assess the psychiatric disorders. The results showed that in the group of the adoptees with over 6 months in preadoption out-of-home care, the likelihood for any psychiatric disorder was significantly increased in HR adoptees (adj. OR 3.12, 95% CI 1.06–9.20) compared to LR adoptees. Among the adoptees with 6 months or less in preadoption out-of-home care, the likelihood for psychiatric disorders was increased in those living in adoptive families with dysfunctional processes (adj. OR 5.09, 95% CI 1.60–16.18). The results of this study indicate that in terms of later mental wellbeing, it is important for children, and especially for children with high genetic risk for schizophrenia spectrum disorders, to have a secure and stable early rearing environment. Particularly, when adoption is needed, the importance of early placement and well-functioning family environment are emphasized.

### Electronic supplementary material

Supplementary tables
