# Supplementary material for: Associations of Duration of Preadoption Out-of-home Care, Genetic Risk for Schizophrenia Spectrum Disorders and Adoptive Family Functioning with Later Psychiatric Disorders of Adoptees
Source: Child Psychiatry Hum Dev. 2022 Aug 13;55(2):350–60. doi: 10.1007/s10578-022-01411-x (PMC10891258; doi:10.1007/s10578-022-01411-x)
Supplement: Supplementary file 1 — Supplementary tables [file 10578_2022_1411_MOESM1_ESM.doc]

**Supplementary material.**

**Table S1. The attrition analysis: The sample used in the analyses (n=171) in comparison with**

**the adoptees not included in the analyses.**

| Characteristics (in parenthesis, number of adoptees with non-missing data) | Total sample of the adoptees (n=382) | | |
| --- | --- | --- | --- |
|  | Study sample  (n=171) | Adoptees not included in the analyses | p-value |
| Genetic risk for schizophrenia spectrum disorders (n=382)  Low risk  High risk | 128 (74.9%)  43 (25.1%) | 64 (30.3%)  147 (69.7%) | <0.001 |
| Family functioning (n=303)  Functional processes  Mildly dysfunctional processes  Dysfunctional processes | 72 (42.1%)  55 (32.2%)  44 (25.7%) | 54 (40.9%)  36 (27.3%)  42 (31.8%) | 0.451 |
| Gender (n=382)  Male  Female | 75 (43.9%)  96 (56.1%) | 107 (50.7%)  104 (49.3%) | 0.182 |
| Time with biological mother in months (n=223)  0 month  1 month or more | 86 (50.3%)  85 (49.7%) | 43 (69.4%)  19 (30.6%) | 0.010 |
| Diagnosed psychiatric disorder (n=382)  Yes  No | 69 (40.4%)  102 (59.6%) | 97 (46%)  114 (54%) | 0.270 |
| Time in preadoption out-of-home care (n=223)  ≤ 6 months,  > 6 months | 90 (52.6%)  81 (47.4%) | 37 (59.7%)  25 (40.3%) | 0.340 |

**Table S2. Bivariate association between the characteristics and psychiatric disorders of the adoptees stratified by the genetic status of the adoptees and the length of pre-adoption out-of-home care (≤ 6 months).**

| Characteristics of the adoptees | HR adoptees 0-6 months  (n=22) | Adoptees with any psychiatric disorder | | | LR adoptees 0-6 months  (n=68) | Adoptees with any psychiatric disorder | | |
| --- | --- | --- | --- | --- | --- | --- | --- | --- |
|  |  | Yes (n=11) | No (n=11) | p-value |  | Yes (n=26) | No (n=42) | p-value |
| Family functioning  Functional processes  Mildly dysfunctional processes  Dysfunctional processes | 12 (54.5%)  5 (22.7%)  5 (22.7%) | 3 (25%)  4 (80%)  4 (80%) | 9 (75%)  1 (20%)  1 (20%) | 0.037 | 29 (42.6%)  20 (29.4%)  19 (27.9%) | 9 (31%)  5 (25%)  12 (63.2%) | 20 (69%)  15 (75%)  7 (36.8%) | 0.028 |
| Gender  Male  Female | 8 (36.4%)  14 (63.6%) | 3 (37.5%)  8 (57.1%) | 5 (62.5%)  6 (42.9%) | 0.375 | 28 (41.2%)  40 (58.8%) | 10 (35.7%)  16 (40%) | 18 (64.3%)  24 (60%) | 0.720 |
| Time with biological mother  in months  0 month  1 month or more | 7 (31.8%)  15 (68.2%) | 2 (28.6%)  9 (60%) | 5 (71.4%)  6 (40%) | 0.170 | 30 (44.1%)  38 (55.9%) | 10 (33.3%)  16 (42.1%) | 20 (66.7%)  22 (57.9%) | 0.460 |

**Table S3. Sensitivity analysis. Associations of the characteristics of the adoptees with the likelihood for psychiatric disorders, by the length of pre-adoption out-of-home care time (≤ 12 months, > 12 months).**

**a) Pre-adoption out-of-home care time ≤ 12 months**

|  | Total n of cases  (n=114) | Adoptees with psychiatric disorders | |  |  | Likelihood for psychiatric disorder | |
| --- | --- | --- | --- | --- | --- | --- | --- |
|  |  | Yes (n=48) | No (n=66) | p-value |  | adj. OR* | 95 % CI |
| Genetic risk for schizophrenia spectrum disorders  Low risk  High risk | 84  30 | 32 (38.1%)  16 (53.3%) | 52 (61.9%)  14 (46.7%) | 0.147 |  | ref. LR  2.04 | 0.80-5.18 |
| Family functioning  Functional processes  Mildly dysfunctional processes  Dysfunctional processes | 49  34  31 | 16 (32.7%)  12 (35.3%)  20 (64.5%) | 33 (67.3%)  22 (64.7%)  11 (35.5%) | 0.012 |  | ref.  1.21  4.15** | 0.46-3.15  1.48-11.66 |
| Gender,  Male  Female | 47  67 | 15 (31.9%)  33 (49.3%) | 32 (68.1%)  34 (50.7%) | 0.065 |  | ref.  2.18*** | 0.95–4.97 |
| Time with biological mother in months  0 month  1 months or more | 48  66 | 16 (33.3%)  32 (48.5%) | 32 (66.7%)  34 (51.5%) | 0.106 |  | ref.  1.23 | 0.52-2.87 |

* Odds ratios (ORs) and 95% CI of OR are based on the logistic regression analysis assessing the likelihood for psychiatric disorder of the adoptees after adjusting for genetic risk, family functioning, gender and time spent with biological mother.

** p < 0.05

*** p < 0.1

**b) Pre-adoption out-of-home care time > 12 months**

|  | Total n of cases  (n=57) | Adoptees with psychiatric disorders | |  |  | Likelihood for psychiatric disorder | |
| --- | --- | --- | --- | --- | --- | --- | --- |
|  |  | Yes (n=21) | No (n=36) | p-value |  | adj. OR* | 95 % CI |
| Genetic risk for schizophrenia spectrum disorders  Low risk  High risk | 44  13 | 13 (29.5%)  8 (61.5%) | 31 (70.5%)  5 (38.5%) | 0.036 |  | ref.  3.93** | 1.02-15.08 |
| Family functioning  Functional processes  Mildly dysfunctional processes  Dysfunctional processes | 23  21  13 | 7 (30.4%)  10 (47.6%)  4 (30.8%) | 16 (69.6%)  11 (52.4%)  9 (69.2%) | 0.436 |  | ref.  2.20  1.11 | 0.59-8.20  0.23-5.38 |
| Gender,  Male  Female | 28  29 | 11 (39.3%)  10 (34.5%) | 17 (60.7%)  19 (65.5%) | 0.707 |  | ref.  0.59 | 0.18–1.99 |
| Time with biological mother in months  0 month  1 months or more | 38  19 | 12 (31.6%)  9 (47.4%) | 26 (68.4%)  10 (52.6%) | 0.244 |  | ref.  1.81 | 0.53-6.12 |

* Odds ratios (ORs) and 95% CI of OR are based on the logistic regression analysis assessing the likelihood for psychiatric disorder of the adoptees after adjusting for genetic risk, family functioning, gender and time spent with biological mother.

** p < 0.05

*** p < 0.1
